# The importance of the multidisciplinary team in the decision-making process of patients undergoing neoadjuvant chemotherapy for breast cancer

**DOI:** 10.1007/s13304-024-01759-w

**Published:** 2024-02-05

**Authors:** Alessandro Fancellu, Valerio Pasqualitto, Pietrina Cottu, Giuliana Giuliani, Lavinia Grasso, Maria Laura Ariu, Alberto Porcu, Valeria Sanna

**Affiliations:** 1 Department of Medicine, Surgery and Pharmacy, Unit of General Surgery 2-Clinica Chirurgica, University of Sassari, Sassari, Italy; 2 AOU Sassari. Unit of Medical Oncology, Sassari, Italy

**Keywords:** Breast cancer, Neoadjuvant chemotherapy, Mastectomy, Breast conserving surgery, Multidisciplinary treatment

## Abstract

**Background and objectives:**

Recent literature suggests that rates of breast conservation surgery (BCS) are lower than expected in patients submitted to neoadjuvant chemotherapy (NAC) for breast cancer. The aim of this study was to underscore the role of the multidisciplinary team (MDT) in the decision-making process of patients who underwent breast surgery after NAC.

**Methods:**

We conducted a retrospective study on patients with breast cancer treated according to an algorithm developed at the Breast Unit of Northern Sardinia between January 2019 and May 2023. Data collected included demographics, tumor characteristics, upfront treatment (surgery or NAC), type of primary surgery (BCS or mastectomy [Ma]) and patients’ adherence to the treatment proposed by the MDT.

**Results:**

Overall, 1061 women were treated during the study period, of whom 164 received NAC (Group A) and 897 upfront surgery (Group B). In group A, conversion from BCS ineligibility to BCS eligibility was observed in 47 patients (40.1%). Final surgery in patients who became BCS-eligible after NAC was BCS in 42 cases (89.3%) and Ma in 5 (10.6%). Rates of patients’ adherence to the treatment proposed by the MDT were significantly better in the Group A (*p* = 0.02).

**Conclusions:**

Our results suggest that the MDT has a pivotal role in increasing the rates of breast conservation in women submitted to NAC.

## Introduction

In recent years, profound innovations aiming to de-escalate treatments in patients with early and advanced breast cancer have been registered. Since a comprehensive approach is essential to improve patient care and outcomes, the multidisciplinary team (MDT) represents a crucial component of patient management, through a strict collaboration among different specialists involved in diagnosis, treatment, and follow-up of breast cancer [1, 2]. In the current scenario, a thorough multidisciplinary discussion based on clinical and biological characteristics of the tumor has the utmost importance in the decision-making process.

Neoadjuvant chemotherapy (NAC) has been increasingly used in the treatment of selected breast cancers, especially in the setting of triple-negative and HER2-positive subtypes [3–5]. One of the scopes of NAC is the conversion to breast conserving surgery (BCS) eligibility of patients who would be candidates to mastectomy (Ma) at the time of diagnosis. NAC regimens, in fact, have the potential of tumor downstaging, thus achieving high rates of complete clinical or pathological response [6, 7]. Nonetheless, rates of BCS after tumor downstaging by induction chemotherapy remain lower than expected [8–10]. The reasons behind the extensive use of Ma in NAC responders are various and also include patient choice and surgeon attitude. However, these findings seem like a contradiction, since the equivalence in terms of oncologic outcomes between BCS and Ma has been demonstrated also in the setting of NAC [11–13].

This article aims to underscore the significance of MDT in breast cancer care, emphasizing its role in increasing the rates of BCT in patients submitted to NAC.

## Materials and methods

### Data source and patient population

**Fig. 1:**
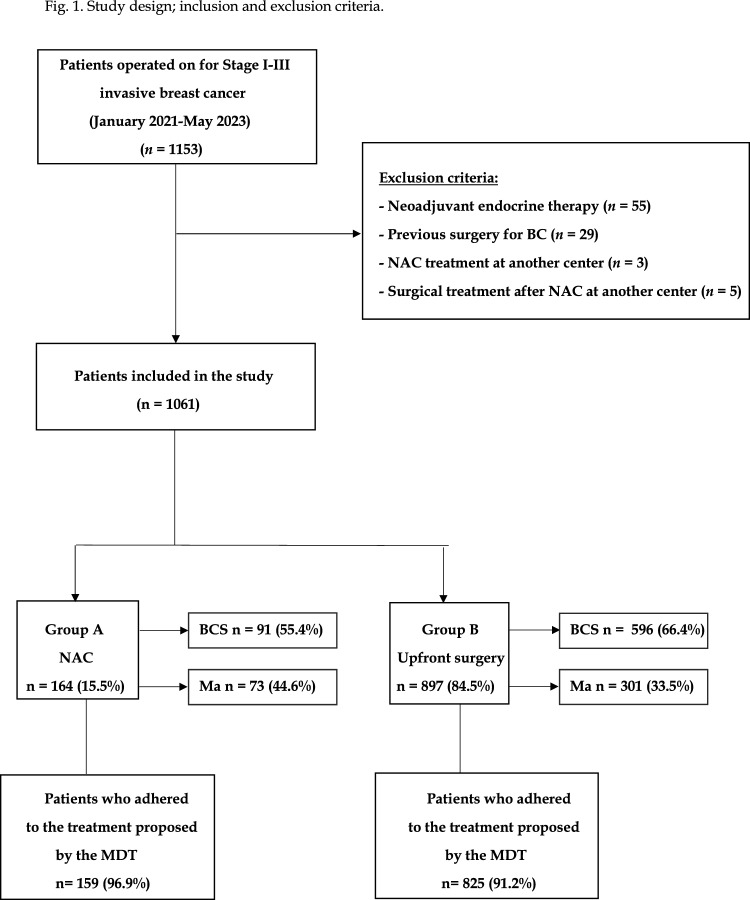
Study design; inclusion and exclusion criteria

**Fig. 2:**
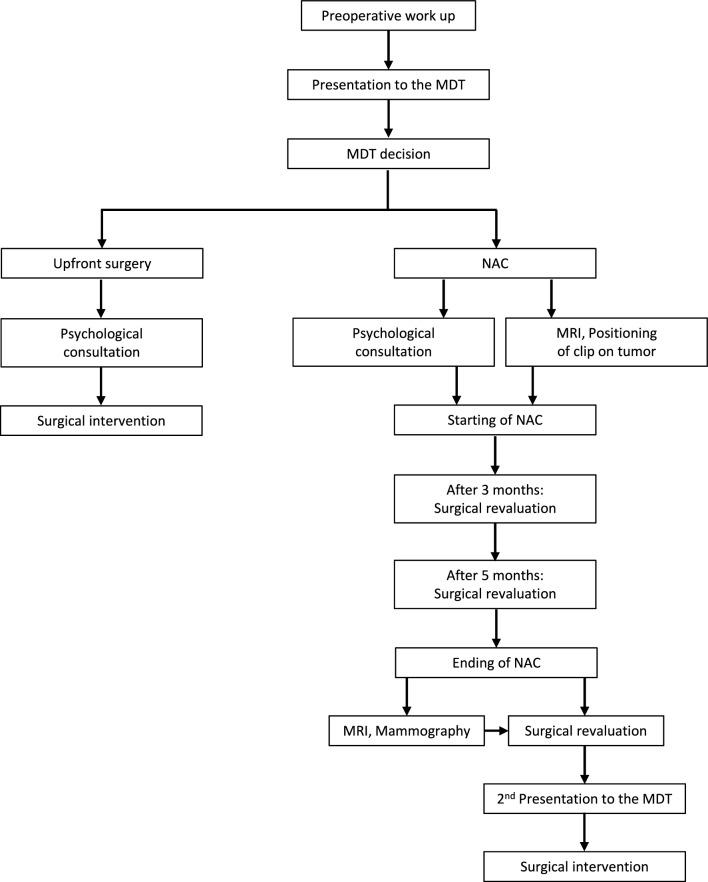
Algorithm for multidisciplinary preoperative management of patients included in the study

We conducted a retrospective review of patients with stage I–III invasive breast cancer who received surgical treatment at the Breast Unit SMAC (Italian acronym for Coordinated Multidisciplinary Corporate Senology) from January 2020 to May 2023. This institution is the referral breast center in the Northern Sardinia, and satisfies the EUSOMA requirements of a specialist breast center [14, 15]. Patients were included if they were followed by the MDT of our Breast Unit during the entire duration of the planned therapeutic program, including surgery, NAC, and radiotherapy, when indicated. Patients submitted to neoadjuvant endocrine therapy were excluded (Fig. 1). Outcomes of patients who received NAC (Group A) were compared with those who received upfront surgery (Group B). The algorithm of management of patients candidates to NAC or upfront surgery is resumed in Fig. 2. Every patient was scheduled to receive a psychological consultation before surgery or starting NAC. In all patients of Group A deemed to undergo a BCS as final surgery, a marker clip was placed using stereotactic or ultrasound guidance. According to the institution algorithm, the breast surgeon was supposed to visit the patients of the NAC group at four different times during the preoperative period. Each member of the MDT was committed to share with the patients the evidences regarding the advantages of BCS over Ma.

For all patients, the following data were extracted: age, menopausal status, tumor size at diagnosis, histological type, tumor grading, molecular subtype, axillary lymph nodes status at final pathology, number of tumors, type of upfront surgery (BCS or Ma), patient adherence to MDT treatment plan. As for patients of the Group A, also the following data were obtained: NAC regimen, tumor response to NAC, proportion of patients obtaining a complete pathologic response, conversion to BCS eligibility among BCS-ineligible patients prior to NAC. Axillary status was evaluated with sentinel node biopsy or axillary lymphadenectomy, when appropriate.

The study was approved by the ethical committee of the Dept of Medicine, Surgery and Pharmacy of the University of Sassari, Italy.

### Statistical analysis

A prospective database was created using Microsoft Office Excel 2019. Quantitative parametric variables were expressed as a mean and SD; qualitative variables, as absolute numbers and percentage. Categorical variables were compared by the chi-square test or the Fisher exact test as appropriate, and continuous variables were assessed by the *t*-test or the Mann–Whitney test. *P* values were two-sided and a *p* value < 0.05 was used as the threshold for statistical significance. Statistical analyses were conducted by using SPSS Statistics 20 (IBM Corporation, USA), and XLSTAT 2016 (Addinsoft Inc, USA).

## Results

### Patient and tumor characteristics

Among 1153 patients with Stage I–III primary breast cancers treated between January 2019 and May 2023, we identified 1061 patients who fulfilled the inclusion criteria. Demographic and tumor characteristics are resumed in Table 1. The mean age at diagnosis was 60.6 (± 12.8) years. The most common histology was invasive ductal carcinoma (82.4%), followed by invasive lobular carcinoma (12.5%). Three hundred and two women (28.4%) were premenopausal. The majority of patients had tumors of grade 2 (69.8%). Overall, the most common molecular subtype was Luminal (66.4%), followed by HER2-positive (18.9%), and triple negative (14.7%). In our cohort, 64.7% of patients underwent BCS, while 35.3% underwent mastectomy as final surgery. Tumor size was greater in women undergoing NAC (Group A) versus those undergoing upfront surgery (Group B) (41.8 mm *vs* 1.9 mm, *p* < 0.0001). Molecular subtype distribution did significantly differ among Group A and Group B (*p* < 0.00001), with triple-negative and HER2-positive subgroup more commonly observed in Group A (29.3% *vs* 12.1% and 48.8% vs 14.5%, respectively), and Luminal subtype more commonly observed in Group B (73.5% vs 21.9%). Lymph nodes positive status was significantly higher in Group A (32.3% vs 16.3% *p* < 0.00001), negative estrogen receptors status (17.4% versus 12.5%, *p* < 0.05), and grading III (19.0% versus 12.8%, *p* < 0.05). Age at diagnosis, menopausal status, and histological subtype did not statistically differ between patients of the two study groups. Patient adherence to MDT treatment plan was significantly higher in the Group A (96.9% vs 91.2%, *p* = 0.023).

**Table 1:** Demographic and tumor characteristics of the study population

Characteristics	Total *n* = 1061	Group A (NAC) *n* = 164	Group B (Upfront surgery) *n* = 897	*p*-value
Age (mean, SD)	60.6 (± 12.8)	58.3 (± 12.3)	61.4 (± 12.4)	0.23
Menopausal status				0.28
Premenopausal	302 (28.4%)	50 (30.5%)	252 (28.1%)	
Postmenopausal	759 (71.6%)	114 (69.5%)	645 (71.9%)	
Tumor size at diagnosis in mm (mean, SD)	2.1 (± 1.8)	41.8 (± 2.3)	1.9 (± 1.5)	< 0.0001*
Histology				0.30
IDC	875 (82.4%)	142 (86.6%)	733 (81.7%)	
ILC	132 (12.5%)	15 (9.1%)	117 (13.0%)	
Other invasive	54 (5.1%)	7 (4.3%)	47 (5.3%)	
Molecular subtype				< 0.00001*
Luminal	705 (66.4%)	36 (21.9%)	669 (73.5%)	
TNBC	156 (14.7%)	48 (29.3%)	108 (12.1%)	
Her2 positive	200 (18.9%)	80 (48.8%)	120 (14.5%)	
Tumour grading				< 0.00001*
1	152 (14.3%)	10 (6.1%)	142 (15.8%)	
2	740 (69.8%)	84 (51.2%)	656 (73.1%)	
3	169 (15.9%)	70 (42.7%)	99 (11.1%)	
Axillary LN status (final pathology)				< .00001*
Positive	257 (24.3%)	53 (32.3%)	146 (16.3%)	
Negative	804 (75.7%)	111 (67.7%)	751 (83.7%)	
Surgical treatment				0.006*
BCS	687 (64.7%)	91 (55.4%)	596 (66.4%)	
Mastectomy	374 (35.3%)	73 (44.6%)	301 (33.5%)	
Patients who followed the treatment proposed by MDT	984 (92.7%)	159 (96.9%)	825 (91.2%)	0.023

*IDC* Invasive ductal carcinoma; *ILC* Invasive lobular carcinoma; *TNBC* triple negative breast cancer; *BCS* Breast conserving surgery; *LN* lymph nodes; *MDT* multidisciplinary team

^*^Statistically significant

### Outcomes of patients of Group A (NAC)

**Fig. 3:**
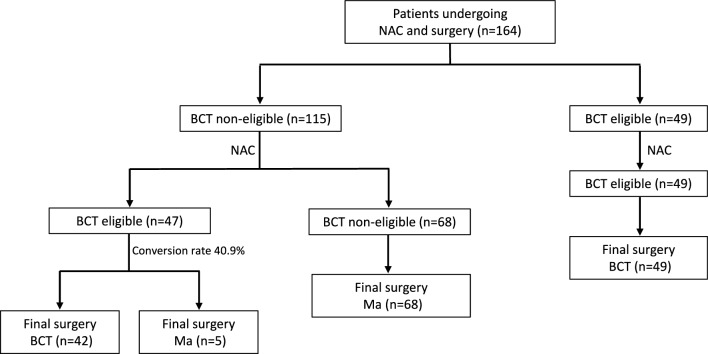
Surgical treatment of patients of Group A (NAC)

One hundred and sixty-four patients received NAC. In 144 of them (88%), a tumor shrinking was seen at clinical and imaging evaluation, while in 20 (12%), a tumor progression or no response occurred. Thirty-three percent of all patients achieved a pathologic complete response after NAC (Table 2). Before NAC, 49 (29.9%) patients were BCS-eligible and underwent BCS as final surgery. Among 115 BCS-ineligible breast cancer patients (70.1%) with potential for downstaging, 47 (40.9%) became BCS-eligible after NAC. Final surgery in these 47 patients BCS-eligible was BCS in 42 cases (89.3%) and Ma in 5 (10.6%) (Fig. 3). Overall, 96.9% of the patients accepted the surgical management planned by the MDT.

**Table 2:** Treatment and outcomes of patients belonging to Group A (NAC group)

	*n* (%)
NAC regimen	
EC + Taxanes	78 (47.6%)
EC + Taxanes + Trastuzumab	80 (48.8%)
Platinum-based regimens	6 (3.6%)
Tumor shrinkage after NAC	144 (88.0%)
Tumor progression or no response during NAC	20 (12.0%)
Pathologic complete response	55 (33.5%)
Conversion to BCS eligibility among BCS-ineligible patients	47 (40.1%)

## Discussion

Our study was designed to evaluate the role of the MDT in the management of patients who undergo NAC and surgery for breast cancer. The main result was that a structured approach involving the different components of the MDT resulted in a high rate of BCS in patients who underwent NAC and surgery, likewise observed in patients who undergo upfront surgery.

BCS followed by radiation therapy represents the most used surgical option in patients with breast cancer, since the equivalence of BCS and Ma in terms of oncologic outcomes has been demonstrated by several trials and retrospective studies. It is noteworthy that the equivalence between BCS and Ma has been shown also in triple-negative and HER2-positive breast cancer, which are the subtypes with the worst prognosis [16, 17]. Several recent evidences showed that patients submitted to BCS and radiotherapy may have even better survival outcomes than those submitted to Ma [18]. It is the so-called concept of “less is more”, that has been leading to a gradual de-escalations of breast cancer treatment [19]. As a consequence, BCS should be considered the gold standard for breast cancer, and Ma should be considered only in patients in whom BCS is not feasible. A recent meta-analysis conducted on more than 1,500.000 patients from 30 studies has shown that patients who underwent BCS had better survival outcomes compared with Ma, and encouraged to include these results in the decision-making process [20]. The advantages of BCS over Ma cannot be overemphasized, and include psychological, sexual and relational benefits correlated to the conservation of the native breast [12, 13, 18, 21].

As regards to the setting of NAC, for years, Ma has been considered as safer than BCS in patients submitted to preoperative systemic treatment, since the latter has been usually reserved to patients with biologically aggressive tumors or with large primary tumors. Consistent with other reports, in our study, about 80 percent of patients submitted to NAC belonged to the triple-negative and HER2-positive subtypes. Data provided by retrospective series suggested that BCS followed by radiation therapy are to be considered a safe approach also in patients submitted to NAC [12, 13, 22, 23]. In a series of 685 patients, local recurrence rates appeared to be low after NAC and BCS, also among BCS patients who had downstaging of their primary tumor after chemotherapy [13]. In a recent study including about 1,500 patients receiving NAC, patients who undergo BCS and radiotherapy had similar 10-year breast cancer-specific survival compared to those who received Ma [12]. In a large population-based study comparing the long-term survival outcomes of patients treated with NAC and BCS plus radiotherapy with patients treated with NAC and Ma, the former group resulted in better disease-free survival and overall survival [23].

One can argue that, in the present study, about 25% of patients having a triple-negative or HER2-positive breast cancer, which are usually commonly considered as good candidates for NAC, were instead submitted to upfront surgery. A possible explanation for that figure may lay in the fact that the use of NAC has increased over the period considered (2019–2023), thus the percentage of patients with aggressive biological behavior scheduled for NAC was higher than 90% during the last year of the study. This increasing trend in the use of NAC is in line with previous reports, given that recommendations about the offer of NAC even in patients with small triple-negative or HER2-positive breast cancer have been applied recently in common practice [24].

In our series, the overall proportion of patients who received NAC and BCS was 55.4%, and the conversion rate from mastectomy to BCT-eligible was 41.1% after NAC. These figures, which were lower than observed in other similar studies, probably reflect the high number of patients with locally advanced breast cancer (the mean tumor size in the NA group was 4.2 cm).

On the other hand, 89.4% of patients who became BCS-eligible after NAC downstaging underwent a BCS as final surgery. This finding is essentially higher than reported in other experiences. In fact, in the literature, the rates of BCS among patients who become BCS-eligible after NAC are lower than expected, especially in relation to the rates of successful response to the pharmacologic treatment [8, 10, 25]. Rates of breast conservation after NAC vary considerably in the literature and range between 13 and 67% [8, 25, 26]. In a recent study including 1383 women with stage I–III breast cancer treated with NAC, among 649 BCS-ineligible, 72% became BCS-eligible after NAC. Of them, only 45% of those < 40 years, 65% of those aged 41–60 years, and 81% of those aged > 61 years choose BCS [9]. In another study with 142 HER2-positive breast cancer patients, more than half of the patients who were BCT-eligible after NAC instead opted for a mastectomy [17].

In line with other reports, we observed that 33% of patients obtained a pathologic complete response after NAC. The high rates of complete pathologic response, particularly seen in the triple-negative subgroup, range from 10 to 74% in recent literature [5, 25–27]. This finding makes it even less understandable why so many patients undergo Ma after NAC, taking into account that one of the main scopes of NAC is to convert patients who are initially ineligible for BCS.

The explanations for the phenomenon of low BCS rates after NAC undoubtedly not only include patient’ choice, but also surgeon’s attitude [10, 25]. We hypostasized that a strategic approach from the MDT as a whole, aiming to give the patient exhaustive information on the current evidences, can help in taking a proper decision-making strategy. In particular, this approach might help in improving the rates of BCS after NAC. In our study, only five patients (3.04%) of the NAC group did not adhere to the surgery planned from the MDT and chose a Ma instead of a BCS. This figure differs slightly albeit in a significant manner from what we observed in the patients of the upfront surgery group (*p* = 0.02).

We believe that the algorithm proposed (illustrated in Fig. 2) may have some points of interest. First, the early psychological consultation, as well as the proper communication of the decision of the MDT meeting, have an important part in the patient understanding of the advantages of breast conservation after NAC. In the literature, there is a lack of consensus on the ideal imaging interval to evaluate the response to NAC, although MRI is considered the most sensitive tool for assessing the response during and at the end of neoadjuvant systemic therapy. Besides, there are no guidelines that address the frequency the surgical assessment of patients undergoing NAC for breast cancer. Every patient undergoing NAC is presented to the MDT at least twice, once prior to start the chemotherapy treatment and a second time prior to surgery. While it is established that a surgical visit is mandatory at the starting and the ending of NAC, some aspects of the management vary according to each institution’s policy. According to our algorithm, the breast surgeon is supposed to visit the patient at least two times during the NAC treatment, other than before and after the chemotherapy treatment, in order to monitor the clinical response and keep sharing the management plan with the patient. We guess that this aspect does play a role in selecting the most appropriate surgical treatment after NAC. To note, we included in the study only those patients submitted to NAC who were followed by our MDT for the entire treatment period, because we believe that the management of a breast cancer patient in a single Breast Unit may increase the quality of cure. The results of our study underscore the relevance of the MDT in breast cancer management.

Treatment outcomes, including the rates of BCS after NAC, are variable and highly surgeon- and center-specific. It is clear that NAC is a highly complex setting in which optimal outcomes are seen in centers that treat patients in a MDT approach [28]. The members of a breast MDT may help in increasing the rates of BCS, by creating a context favorable to breast-conservation based on recent scientific evidences. Nonetheless, an optimal treatment strategy requires a shared approach between patient and clinician. Once an appropriate decision has been taken by the MDT, it should be taken into account that the final decision on surgical treatment is highly dependent on individual patient preferences.

We recognize that our manuscript has some limitations, the main being the retrospective design and the small sample size. Besides, no data on the treatment of the axillary basin were extracted and discussed. However, the study was designed to specifically investigate the rates of BCS and Ma as surgical approach after preoperative chemotherapy. Despite these limitations, it is one of the few studies in which the issue of the surgical management of patients submitted to NAC has been addressed from the perspective of the MDT approach. An algorithm has been proposed, that might serve for improving the strategies of management of patients submitted to NAC.

## Conclusions

Despite improved response to NAC, rates of BCS in this setting remain suboptimal in recent trials. As breast cancer treatment becomes more precise, a close interaction between the breast specialists may help to determine the optimal management strategies. Our results suggest that the MDT has a pivotal role in increasing the rates of breast conservation in women submitted to NAC.
